# Integrated transcriptome and metabolome analysis reveals the molecular mechanism underlying differences in Psa resistance between *Actinidia valvata* and *Actinidia chinensis*

**DOI:** 10.3389/fpls.2026.1724662

**Published:** 2026-02-24

**Authors:** Rongxiang Zhu, Jianyou Gao, Cuixia Liu, Liming Xia, Kaiyu Ye, Beibei Qi, Jiewei Li, Faming Wang

**Affiliations:** Guangxi Key Laboratory of Plant Functional Phytochemicals and Sustainable Utilization, Guangxi Zhuang Autonomous Region, Guangxi Institute of Botany, Chinese Academy of Sciences, Guilin, China

**Keywords:** integrated transcriptome-metabolome, kiwifruit, lignin biosynthesis, plant resistance, *Pseudomonas syringae* pv. *actinidiae*

## Abstract

Kiwifruit has high economic value, but is susceptible to bacterial canker disease caused by *Pseudomonas syringae* pv. *actinidiae* (Psa). To dissect the resistance mechanisms, we performed an integrated transcriptomic and metabolomic analysis of a resistant species, *Actinidia valvata*, and a susceptible cultivar, *A. chinensis* ‘Hongyang’ (HY), following Psa infection. After Psa inoculation, a total of 1781 differentially expressed genes (DEGs) were identified collectively in HY and *A. valvata*, which were mainly annotated to 20 pathways, including plant-pathogen interaction, MAPK signaling pathway, and plant hormone signal transduction. Besides, 964 differentially accumulated metabolites (DAMs) were detected collectively in the two varieties, with 369 up-regulated and 595 down-regulated metabolites showing significant changes post-infection. Notably, flavonoids, phenolic acids, amino acids and alkaloids were the dominant DAMs, with *A. valvata* specifically accumulating key lignin-related metabolites (L-phenylalanine), while HY exhibited a net downregulation of most metabolites. DEGs and DAMs were co-enriched to 25 metabolic pathways, among which biosynthesis of various plant secondary metabolites was prominent. Key genes in the phenylpropanoid biosynthesis pathway were identified, genes related to lignin synthesis, including cinnamoyl-CoA reductase (CCR), cinnamyl alcohol dehydrogenase (CAD), and Peroxidase (POD), were significantly up-regulated in *A. valvata*, and their high expression levels correlated with reduced accumulation of lignin intermediates and elevated production of mature lignin polymers in *A. valvata*. This indicates that *A. valvata* likely contributes to enhanced lignin synthesis to defend against Psa infection, compared with HY. The results may elucidate the metabolic networks and molecular mechanisms of kiwifruit in response to bacterial canker disease.

## 1 Introduction

Kiwifruit (*Actinidia* spp.) is a globally valued fruit crop renowned for its unique flavor, polyphenolic compounds, and nutritional benefits (vitamin C and intestinal health promotion). Most important commercial cultivars worldwide belong to *Actinidia chinensis* (including var. *chinensis* and *deliciosa*), among which ‘Hongyang’ (HY) is a representative susceptible cultivar to bacterial canker, one of the most destructive diseases caused by Psa (Wang et al., 2023a). In contrast, species from Sect. Leiocarpae (*Actinidia valvata* Dunn, *Actinidia arguta*, *Actinidia macrosperma* and *Actinidia kolomikta*) exhibit high resistance to Psa (Wang et al., 2020).

Over the last few years, bacterial canker caused by Psa has emerged as a global threat to kiwifruit orchards, seriously undermining the booming kiwifruit industry and compromising fruit yield and quality (Scortichini et al., 2012; Vanneste, 2017). This bacterium comprises four major biovars, designated Psa1 to Psa4, among which biovar 3 is responsible for the worldwide outbreak of kiwifruit bacterial canker (Vanneste, 2017; Zhao et al., 2019). Basal resistance to Psa was suppressed in kiwifruit at cool growth temperature (16°C) compared with at normal temperature (24°C) (Wu et al., 2025). The control of kiwifruit’s bacterial canker may only rely on preventive methods since there is no curative treatment known for Psa. Preventive chemical applications can help contain spread but are ineffective alone, requiring complementary orchard hygiene and field management to reduce inoculum (Santos et al., 2024). Current control options are limited, with field practices dominated by copper-based compounds and antibiotics sprays and smears (Cameron and Sarojini, 2014; Liu et al., 2021). These substances, however, raise environmental concerns and cause bacterial resistance, phytotoxicity, and environmental persistence (Altimira et al., 2012; Cameron and Sarojini, 2014). Thus, alternative methods are needed to balance sustainable agriculture, environmental protection, and reduced antibiotic/metal-resistant bacteria. Exploring the intrinsic resistance mechanisms of *A. valvata* and translating them to susceptible cultivars is urgent.

Genetic engineering has emerged as a promising approach in recent years. With the continuous advancement and refinement of kiwifruit genome sequencing (Huang et al., 2013; Lu et al., 2024; Wu et al., 2019), gene functions have been further explored, making the application of resistance genes a novel strategy to enhance kiwifruit’s resistance against bacterial canker. Through Weighted Gene Co-Expression Network Analysis (WGCNA), *AcC3H1* and *AcREM14* were identified in two materials with significantly different Psa resistance (which were obtained from a hybrid population of *A. chinensis* var. *chinensis*), and exhibited high expression levels in the highly resistant hybrid material upon Psa infection. Further overexpression of *AcC3H1* and *AcREM14* in kiwifruit enhanced disease resistance by upregulating enzyme activities in the salicylic acid (SA) signaling pathway and activating the expression of related disease-resistant genes (Zhao et al., 2024). The kiwifruit NAC transcription factor *AcNAC10*, which is upregulated in resistant variety RH12 upon Psa infection, and is activated by *AcTGA07* and acts as a transcriptional repressor of *AcLOX3*, and they elucidated the “*AcTGA07*-*AcNAC10*-*AcLOX3*” transcriptional cascade that enhances kiwifruit resistance to Psa by inhibiting jasmonic acid (JA) synthesis (Zhao et al., 2025). In recent years, an increasing number of resistance genes have been reported, such as kiwifruit *AcLac35* (Li et al., 2025), *AcJAZ2L2* (Wang et al., 2025), rice *SPR9* (He et al., 2023), *OsBDR1* (Wang et al., 2023b), and pear *PbrChiA* (Chen et al., 2023). Therefore, on one hand, transferring heterologous resistance genes into highly susceptible main cultivars can generate transgenic lines with significantly reduced susceptibility to bacterial canker; on the other hand, enhancing plant resistance can be achieved by increasing lignin content in cane tissues.

Upon Psa infection, kiwifruit initiates a two-tiered innate immune system to counteract pathogen invasion, consisting of pattern-triggered immunity (PTI) and effector-triggered immunity (ETI). PTI, as the first line of defense, is activated when cell surface-localized pattern recognition receptors (PRRs), often belonging to the receptor kinase (RK) family, such as flagellin-sensitive 2 (FLS2), chitin elicitor receptor kinase 1 (CERK1), and microtubule-associated protein 2 (MAP2), recognize conserved pathogen-associated molecular patterns (PAMPs) of Psa (Qin et al., 2022; Wang et al., 2018). This recognition triggers downstream responses including stomatal closure and activation of early defense-related genes, which collectively restrict initial pathogen colonization. To overcome PTI, successful Psa strains secrete effector proteins via the bacterial type III secretion system (T3SS) to interfere with plant immune signaling and enhance virulence. In turn, kiwifruit activates ETI, the second immune layer, which relies on intracellular resistance (R) genes (rar1, sgt1, hsp90a, rpm1) to specifically recognize these effectors (Qin et al., 2022; Wang et al., 2018). ETI typically elicits a stronger and more specific defense response, often accompanied by hypersensitive response (HR) and programmed cell death (PCD) at the infection site to limit pathogen spread. Notably, PRRs (as core components of PTI) and RKs (as critical mediators of PAMP recognition) play indispensable roles in bridging PTI activation and subsequent ETI crosstalk, making their expression dynamics key indicators of the kiwifruit’s ability to mount effective immune responses against Psa.

A critical defense against Psa is the plant cell wall (Wan et al., 2021), where lignin (a major component of secondary walls) acts as a physical barrier to pathogen invasion. Lignin biosynthesis involves a cascade of enzymes, including phenylalanine ammonia-lyase (PAL), cinnamate 4-hydroxylase (C4H), 4-coumarate-CoA ligase (4CL), p-coumarate 3-hydroxylase (C3H), caffeic acid O-methyltransferase (COMT), CCR, and CAD. CCR and CAD reduce the precursors to monomers such as p-coumaryl alcohol, coniferyl alcohol, and sinapyl alcohol. After these monomers are transported to the cell wall, they need to undergo oxidative polymerization to form lignin macromolecules. POD plays a crucial role in this process, in which the monomers eventually polymerize into lignin polymers. Prior studies in kiwifruit have linked lignin accumulation to Psa resistance. The laccase gene *AcLac35*, which critical for lignin polymerization enhances cell wall integrity, overexpressing *AcLac35* increases lignin content by 30% and reduces Psa-induced lesion area by 45%, with its expression activated by the transcription factor *AcSPL9* (Li et al., 2025). The Psa-resistant *Actinidia eriantha* genotype Eri-1 activates PTI and ETI in leaves upon Psa inoculation but suppresses downstream HR signaling to limit ROS and PCD, while predominantly activating lignin biosynthesis genes to accumulate lignin, which confines and eliminates Psa, revealing Eri-1’s unique leaf resistance mechanism to Psa (Gao et al., 2025).

Metabolites are proximal to plant phenotypes and provide direct evidence for functional defense responses against pathogens. However, most existing studies on kiwifruit-Psa interactions have focused on single resistance-related pathways or leaf-specific resistance mechanisms (Gao et al., 2025; Li et al., 2025). with limited attention paid to genotype-specific metabolic reprogramming and its link to cane resistance, the primary infection site of Psa. Crucially, systematic integrated transcriptomic and metabolomic analyses to bridge metabolic differences, transcriptional regulation, and resistance/susceptibility mechanisms remain lacking.

To address these gaps, we Built on prior screenings of kiwifruit genotypes with contrasting resistance levels to bacterial canker (Wang et al., 2023a), we selected 5 days post inoculation (dpi) as the sampling time point for the present study, which is a critical transition stage from initial infection to pathogen expansion that enables the effective capture of resistant-susceptible differences in molecular and metabolic responses (McAtee et al., 2018; Song et al., 2019). The present study conducts comparative transcriptomic and metabolomic analyses of two cultivars with distinct resistance phenotypes, *Actinidia valvata* Dunn (*A. valvata*, resistant) and *Actinidia chinensis* (HY, susceptible), which subjected to both Psa inoculation and water treatment (control). This work aims to decipher the intricate metabolic pathways and gene regulatory networks associated with disease resistance, with a focus on identifying key metabolites and genes. This comparative framework fills the gap in understanding interspecific resistance differences at the multi-omics level.

Such an analysis will not only facilitate the identification of Psa-responsive molecular networks in kiwifruit, but also deepen our understanding of disease resistance mechanisms in fruit trees, ultimately accelerating the breeding of bacterial canker-resistant kiwifruit cultivars.

## 2 Materials and methods

### 2.1 Kiwifruit materials and the preparation of bacterial inoculum

*A. valvata* and HY were preserved in the *Actinidia* germplasm repository of the Guangxi Institute of Botany (GXIB) in Guilin, China.

The Psa strain GxL01 used in this study was Psa3, which is the most virulent biovars to *Actinidia* (Scortichini et al., 2012). For convenient subsequent bacterial tracking and microscopic visualization, a GFP-tagged derivative of the wild-type Psa3 strain was used in this experiment. This tagged strain was preserved in our laboratory and constructed via vector-mediated transformation with the PDSK-GFPuv plasmid (Gao et al., 2025). The bacteria were cultured on Luria Broth (LB) solid medium at 25°C for 48 h. Then, a single colony was picked and transplanted to 50 mL LB liquid medium. The bacterial inoculum was incubated at 25°C for about 48 h under shaking; the bacterial suspension was centrifuged at 4000 rpm for 5 min, and the concentration of the final bacterial suspension was adjusted to 1×10^9^ colony forming units (CFU/mL) for use in the *in vitro* inoculation experiment.

### 2.2 *In vitro* cane‐inoculation assay

Cane inoculation with Psa was performed following the method described by Wang et al. (2019). During the winter dormancy stage, mature woody canes (approximately 30 cm in length) were selected for the assay. Each treatment group included 5 individual cane samples, with 3 independent biological replicates. At the midpoint of each cane, a 0.5 cm diameter wound was created, and then 20 μL of Psa suspension with a concentration of 10^9^ CFU/mL was applied to the wounded site. Once the inoculum had been fully absorbed, the canes were placed in a draining tray with two layers of pre-moistened sterile absorbent paper. Two additional layers of pre-moistened sterile filter paper were then placed on top of the canes to maintain moisture and ensure the wound facing upward. The trays were subsequently transferred to a growth chamber, where the environment was maintained at 12 ± 2°C and 90% relative humidity, and incubated for 5 days. This time point was selected because it represents a critical transition stage from initial Psa colonization to active pathogen expansion, during which the molecular and metabolic differences between resistant and susceptible genotypes are sufficiently pronounced to be effectively captured (Song et al., 2019). The bark was peeled off the canes to observe phenotypic changes and measure the length of lesions caused by Psa infection, phloem tissue isolated after bark removal from canes was uniformly used for RNA-seq, metabolomic analysis, and lignin content determination. Psa bacterial suspension was used to treat the canes of *A. valvata* and HY, with the corresponding samples named *A. valvata*_Psa and HY_Psa; meanwhile, sterile water was used to treat the canes of the two materials, which served as the mock control for the Psa-inoculated group, and the control samples were named *A. valvata*_ck and HY_ck.

### 2.3 Detection and analysis of transcriptome

Total RNA was extracted from cane samples of both the resistant *A. valvata* and the susceptible HY plants which inoculation with Psa and water, using the OminiPlant RNA Kit (DNase I) (ComWin Biotech Co., Ltd.). The RNA was precipitated with ethanol, collected by centrifugation, and dissolved in DEPC water. The NanoDrop 2000 spectrophotometer (Thermo Fisher Scientific) was used to determine RNA purity via A260/A280 and A260/A230 ratio measurements. RNA integrity was further verified by 1.2% agarose gel electrophoresis, where clear and intact 28S and 18S rRNA bands were observed, confirming the high quality of the isolated RNA.

Transcriptomic analysis of kiwifruit in response to Psa infection and water treatment at 5 dpi was conducted by BGI Genomics Co., Ltd. (Shenzhen, China). using the DNBSEQ-T7 sequencing platform (MGI Tech Co., Ltd., Shenzhen, China), a high-throughput platform based on DNA Nanoball (DNB) core technology. Strand-specific cDNA libraries (paired-end, PE) were constructed following BGI’s standard protocol: briefly, mRNA was enriched from total RNA using oligo(dT) magnetic beads, fragmented into ~200 bp fragments, reverse-transcribed into cDNA with random hexamers, and then subjected to end repair, A-tailing, adapter ligation, and PCR amplification (12–15 cycles). The constructed libraries were quantified using a Qubit 3.0 Fluorometer (Thermo Fisher Scientific) and validated for insert size via Agilent 2100 Bioanalyzer before sequencing. DNBs were generated by rolling-circle amplification of the cDNA libraries, and sequencing was performed with a read length of 2 × 150 bp (PE150) to ensure high data quality and coverage. Each of the 4 treatment groups was set with 3 independent biological replicates, and a total of 12 cDNA libraries (4 groups × 3 replicates) were constructed to guarantee the statistical robustness of the transcriptomic data. Raw sequencing data were preprocessed using fastp v0.23.2 (a high-efficiency tool preferred in BGI’s pipeline) to remove adapter sequences, low-quality reads (Phred quality score < 20), reads with N content > 5%, and short reads (< 50 bp) to obtain clean reads. The clean reads were then aligned to the publicly available *A. valvata* whole-genome reference sequence (with the accession number PRJNA1169670.) using HISAT2 v2.2.1 (a splice-aware aligner commonly used in BGI’s analysis) with default parameters except for setting ‘--dta-cufflinks’ to optimize alignment for transcript assembly.

Gene expression abundance was quantified using StringTie v2.2.1 (consistent with BGI’s standard gene-level quantification workflow) combined with HTSeq-count v0.13.5. The final gene-level expression values were calculated as FPKM (Fragments per kilobase of transcript per million fragments mapped). For differential expression analysis, raw read count data generated by HTSeq-count v0.13.5 were imported into the DESeq2 R package (v1.40.2) without prior normalization, as DESeq2 incorporates an internal normalization step to eliminate systematic biases between samples.

Differentially expressed genes (DEGs) were identified using the DESeq2 R package (v1.40.2), a tool integrated in BGI’s differential expression analysis pipeline, with the following workflow: first, gene-level count data from HTSeq-count were imported into DESeq2; then, the software was used to model gene expression with treatment group as the main factor, and DEGs were filtered based on the statistical thresholds of |log2 fold change (FC)| ≥ 1 and adjusted *P*-value (*P*adj) < 0.05. The Benjamini-Hochberg method was employed for multiple testing correction to control the false discovery rate (FDR), a standard practice in BGI’s transcriptomic analysis.

Gene Ontology (GO) enrichment analysis was performed using GOseq R package software, and Kyoto Encyclopedia of Genes and Genomes (KEGG) signaling pathway enrichment analysis was performed using KOBAS software (V3.0) with a significance threshold of *P*adj < 0.05.

### 2.4 Metabolite analysis by ultra-performance liquid chromatography-tandem mass spectrometry

Metabolomic profiling of kiwifruit samples, including *A. valvata* and HY under Psa-inoculated and mock conditions, were performed by MetWare Biotechnology Co., Ltd. (Wuhan, China), using its standardized widely targeted metabolomics protocol. The experimental materials were cane tissues of *A. valvata* (resistant) and HY (susceptible) under Psa-inoculated and mock (water) conditions, which were the same batch of biological materials as those used for RNA-seq analysis. The sampling time point was consistent with that of RNA-seq (5 dpi). Briefly, 100 mg of fresh kiwifruit tissue was frozen in liquid nitrogen, ground into powder, and extracted with pre-cooled 70% (v/v) methanol (containing 0.1% (v/v) formic acid) via vortexing, 4°C sonication, and centrifugation (12,000 × g, 15 min, 4°C); the supernatant was filtered through a 0.22 μm organic-phase filter before UPLC-MS/MS analysis. UPLC separation was performed on an Agilent 1290 system with a Waters ACQUITY UPLC HSS T3 column (2.1 mm × 100 mm, 1.8 μm) using a gradient elution of 0.1% formic acid in water (solvent A) and acetonitrile (solvent B); MS/MS detection was carried out on a QTRAP 6500+ spectrometer (AB Sciex) in ESI ± modes with multiple reaction monitoring (MRM) based on MetWare’s in-house metabolome database (MWDB v4.0). Raw data were quantified via Analyst 1.6.3 software; multivariate analysis (PCA, OPLS-DA) was done using SIMCA 14.1, and differential metabolites were screened by criteria: VIP > 1 (from OPLS-DA), fold change (FC) ≥ 2 or ≤ 0.5, and *P* < 0.05 (Student’s t-test), followed by functional annotation via the KEGG database.

### 2.5 Determination of lignin content

Lignin content was measured using the Lignin Content Detection Kit (MZS-2-G; Suzhou Comin Biotechnology Co. Ltd., Suzhou, Jiangsu, China), following the manufacturer’s recommended protocol. Each sample was analyzed with 3 biological replicates. The experimental materials were the same batch of kiwifruit cane tissues as those used for RNA-seq and metabolomic analysis. Lignin content was calculated using a standard curve generated with the standard substance provided in the kit, expressed as mg/g fresh weight (FW).

### 2.6 Prediction of Cis-acting elements in promoters

To predict cis-acting regulatory elements, promoter sequences (2000 bp upstream of the transcription start site, TSS) of target genes were extracted using TBtools (v1.120) with the “Sequence Extraction” module: the genome sequence file (FASTA format) and gene annotation file (GFF3 format) of *A. valvata* were imported, and the parameter “Upstream length from TSS” was set to 2000 bp, while “Downstream length from TSS” was set to 0 bp to exclude downstream sequences. Sequences containing ambiguous bases (N content > 5%) or incomplete length (due to gene location at chromosome ends) were filtered out to avoid interference with prediction results. Cis-acting regulatory elements were predicted via the plant-specific database PlantCARE (https://bioinformatics.psb.ugent.be/webtools/plantcare/html/) with default parameters (E-value ≤ 1e-5, minimum match length ≥ 8 bp). Elements associated with defense response and secondary metabolism (e.g., W-box, MYB-binding site) were retained for further analysis.

### 2.7 RT-qPCR analysis

Total RNA was isolated using an OminiPlant RNA Kit (DNase I) (ComWin Biotech Co., Ltd.), followed by first-strand cDNA synthesis for RT-qPCR using TranScript^®^ One-Step gDNA Removal and cDNA Synthesis Supermix (TransGen Biotech Co., Ltd). RT-qPCR analysis was performed using LightCycler^®^ 480 SYBR Green I Master mix (Roche) according to the manufacturer’s protocol. The kiwifruit *Actin* gene was used as the internal control for normalization of gene expression. All primers are listed in **Supplementary Table S9**. Each assay contains 3 biological replicates.

### 2.8 Statistical analysis

Statistical analyses were performed using GraphPad Prism version 8.0. All data from the present study are presented as the mean ± SEM. Student’s t-test was used for two-group comparisons, with significance indicated by asterisks (** P < 0.0001). For multiple comparisons, one-way ANOVA followed by Tukey’s multiple comparison test was applied, and differences were considered statistically significant at P < 0.05.

## 3 Results

### 3.1 Changes in symptoms of *A. valvata* and HY shoots infected with Psa

**Figure 1:**
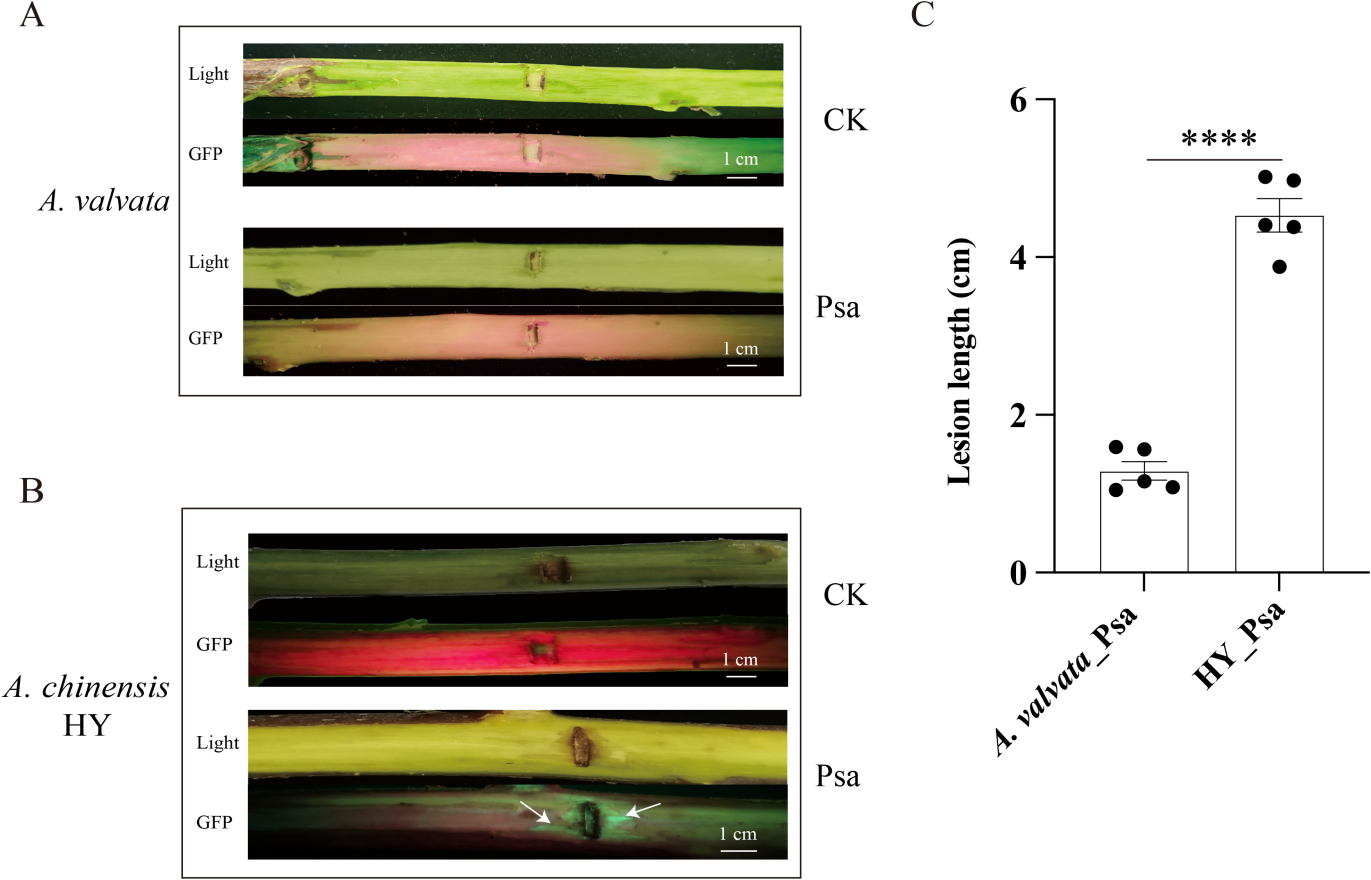
Phenotypic observation and physiological data determination of kiwifruit after Psa inoculation (*A. valvata* and HY). **(A)** White light and fluorescence phenotypes of *A. valvata*_ck and *A. valvata*_Psa. **(B)** White light and fluorescence phenotypes of HY_ck and HY_Psa; lesion locations are indicated by arrows. **(C)** Lesions length of *A. valvata* and HY at 5 days after Psa inoculation. Each treatment group included 5 individual cane samples, with 3 independent biological replicates. CK, Control; Psa, *Pseudomonas syringae* pv. *actinidiae*. The red color in CK groups originates from plant endogenous components. Without GFP-derived green fluorescence to mask these signals, CK groups exhibit red. The green color at infection sites (marked by white arrows) in Psa3-GFP inoculated groups is emitted by heterologously expressed Green Fluorescent Protein (GFP) in modified Psa3—GFP emits intense green fluorescence upon excitation by 488 nm blue-violet light. ****P < 0.0001.

Different kiwifruit varieties exhibit varying degrees of resistance to Psa. In this study, *A. valvata* and HY were selected to observe phenotypic changes after Psa inoculation. The canes of sensitive and non-sensitive varieties showed different lesion lengths after inoculation with Psa (**Figures 1A, B**). The average length of waterlogged lesions on the canes of *A. valvata*, which had high resistance to Psa, is 12.89 mm. On the contrary, HY with 45.31 mm (average) waterlogged lesions, shows a higher susceptibility to Psa (**Figure 1C**). These results indicate that different varieties of kiwifruit have different resistance to Psa, with HY being highly susceptible and *A. valvata* being highly resistant. But the molecular basis underlying the high resistance of *A. valvata* to Psa requires further research.

### 3.2 Determination of DEGs and transcriptomic analysis of canes inoculation with Psa

To elucidate the molecular mechanisms of kiwifruit’s resistance to Psa, high-throughput RNA-Seq analysis of 12 cDNA libraries of canes was performed. After data cleaning and quality control, a total of 76.68 GB of clean data were generated and the clean reads ranged from 6.32×10^7^ to 6.48×10^7^. The Q30 value of each library exceeded 92.09% (**Supplementary Table S1**). The clean reads were obtained from RNA-seq raw data by filtering out uncertain reads, adapter related reads, and low-quality reads. These high-quality reads guaranteed the further gene expression analysis. Clean reads from each sample were mapped to the designated reference genome(*A. valvata*).The mapping rate ranged from 80.93% to 92.83% (**Supplementary Table S1**).

**Figure 2:**
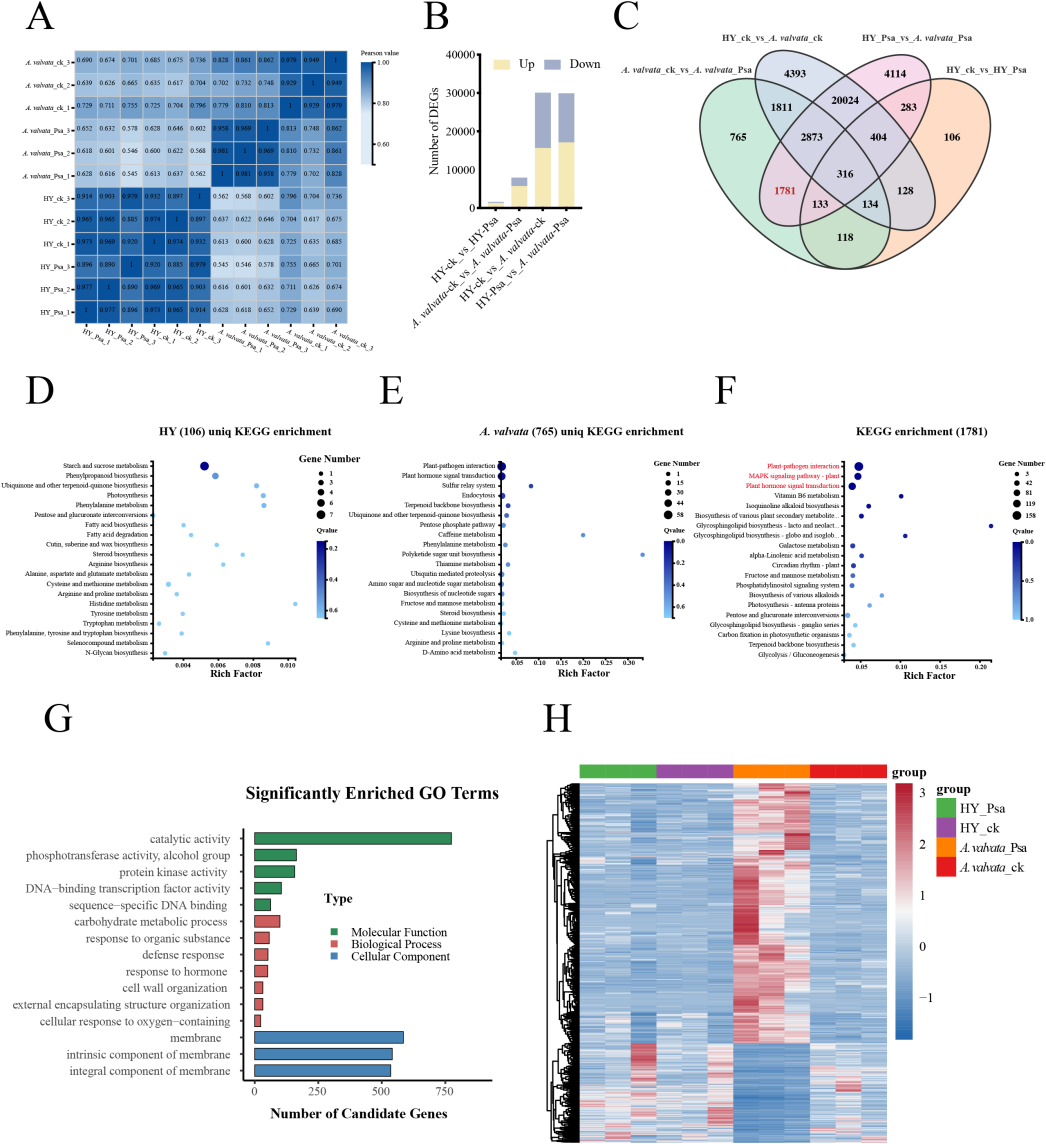
Preliminary analysis of transcriptomic data. **(A)** Pearson correlation heatmap of transcriptome samples. The color intensity represents the magnitude of the correlation coefficient, with darker colors indicating higher similarity in gene expression patterns among samples. **(B)** Number of DEGs in the four comparison groups. **(C)** Venn diagrams showing the overlap of DEGs among different treatments. Each circle represents one comparison group, and overlapping regions indicate the number of common DEGs between groups. **(D)** KEGG enrichment analysis of 106 DEGs in HY. **(E)** KEGG enrichment analysis of 765 DEGs in *A. valvata*. **(F)** KEGG pathway enrichment analysis of 1781 DEGs. **(G)** GO enrichment analysis of 1781 DEGs. **(H)** Heatmap of 1781 intersecting DEGs. The color gradient (from blue to red) indicates expression levels from low to high, reflecting the clustering and differential expression patterns of these core DEGs across samples. DEGs, Differentially Expressed Genes; KEGG, Kyoto Encyclopedia of Genes and Genomes; GO, Gene Ontology.

Unsupervised correlation analysis revealed that biological replicates from the same experimental group under identical treatment conditions exhibited high similarity, demonstrating consistent gene expression patterns across samples and validating the reliability and reproducibility of the transcriptome data (**Figure 2A**). In the HY_ck_vs_ HY_Psa comparison, 1313 up-regulated and 309 down-regulated; in the *A. valvata*_ck_vs_*A. valvata*_Psa were 7931 DEGs (5741 up-regulated and 2190 down-regulated); in the HY_ck_vs*_A. valvata*_ck comparison, there were 30083 DEGs (15635 up-regulated and 14448 down-regulated); and HY_Psa_vs_*A. valvata*_Psa comparison, there were 29928 DEGs (17118 up-regulated and 12810 down-regulated); (**Figure 2B**). Transcriptomic analysis revealed a striking difference in transcriptional activation intensity between the two genotypes, *A. valvata* exhibited a robust defense-related transcriptional response (approximately 5-fold more DEGs than HY), while HY showed a weak and limited response, which may underpin their resistance divergence.

We constructed a Venn diagram to show the overlaps of DEGs among the four group (**Figure 2C**). 316 DEGs were shared among the four group. 133 DEGs were shared among the *A. valvata*_ck_vs_*A. valvata*_Psa, HY_Psa_vs_*A. valvata*_Psa and HY_ck_vs_HY_Psa. There were 765 DEGs specifically present in the comparison between *A.valvata*_ck and *A. valvata*_Psa, whereas only 106 DEGs were specifically identified in HY_ck vs HY_Psa. A total of 1781 DEGs were shared between the comparison groups *A. valvata*_ck_vs_*A. valvata*_Psa and HY_Psa_vs_*A. valvata*_Psa, which were important candidate genes (**Figure 2C**; **Supplementary Table S2**).

To explore the differential responses of two kiwifruit species to Psa infection, we analyzed the KEGG enrichment of DEGs in three groups: 106 DEGs unique to HY, 765 DEGs unique to *A. valvata*, and 1781 DEGs shared between *A. valvata*_ck vs *A. valvata*_Psa and HY_Psa vs *A. valvata*_Psa comparisons. For HY, the 106 specific DEGs were exclusively enriched in basic metabolic pathways (starch and sucrose metabolism, phenylpropanoid biosynthesis), with no significant enrichment in defense-related signaling pathways (**Figure 2D**). In contrast, *A. valvata* exhibited a defense-oriented enrichment profile: The 765 A*. valvata*-specific DEGs were specifically enriched in two core defense pathways: Plant-pathogen interaction and Plant hormone signal transduction (**Figure 2E**). The 1781 A*. valvata*-core shared DEGs were significantly enriched in three pivotal defense-related pathways: Plant hormone signal transduction, MAPK signaling pathway, and Plant-pathogen interaction, which showing high overlap with the enrichment pathways of *A. valvata*-specific DEGs (**Figure 2F**).

Further, GO term enrichment analysis of 1781 DEGs classified them into three functional groups: biological processes, cellular components, and molecular functions (**Figure 2G**), in the biological processes category, the DEGs were mainly enriched in “carbohydrate metabolic process”, “cell communication”, and “response to chemical”; in the molecular functions category, the DEGs were mainly enriched in “catalytic activity”; and in the cellular component category, the DEGs were mainly enriched in “membrane”, “intrinsic component of membrane” and “integral component of membrane”. And the expression levels of 1781 DEGs were analyzed, showed the most of DEGs were upregulated in *A. valvata* post-Psa inoculation (**Figure 2H**), including three pathogenesis-related (PR) proteins and 29 resistance receptor kinases (RKs), which are known to mediate plant immunity (**Supplementary Table S3**).

### 3.3 Comparison of differentially accumulated metabolites in kiwifruit canes

**Figure 3:**
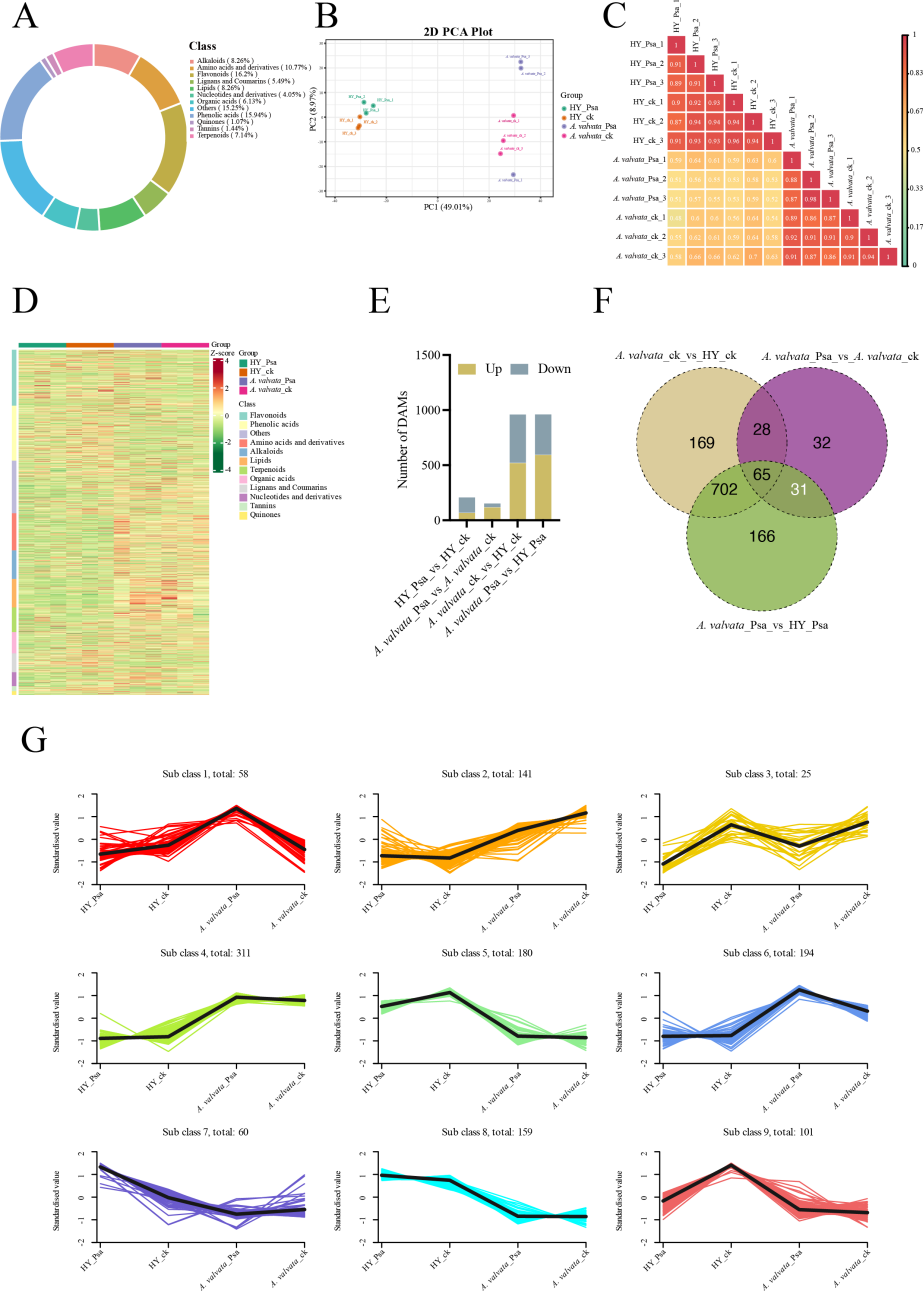
Analysis of metabolome changes in *A. valvata* and HY under Psa infection. **(A)** Summary of metabolites: The ring chart shows the composition of detected metabolites and their different classes. **(B)** Principal component analysis of all metabolites from different materials and treatments. PC1 and PC2 represent the first and second principal components, respectively. **(C)** Correlation analysis of test samples. The heatmap shows the metabolite correlation between different samples, with color depth indicating the correlation intensity (the darker the red, the higher the positive correlation). **(D)** Hierarchical clustering heatmap of DAMs between the two materials after Psa infection. The heatmap shows the expression patterns of differential metabolites, and color indicates the relative abundance of metabolites (red = up-regulation, green = down-regulation). **(E)** Statistics of up-regulated and down-regulated DAMs in the two materials after Psa and water treatments. **(F)** Venn diagrams showing differential metabolites in the two materials after Psa and water treatments. The diagram shows the number of common and unique differential metabolites between the two materials. **(G)** K-means cluster analysis of metabolites in *A. valvata* and HY. The X-axis represents different treatments, and the Y-axis represents the standardized Z-score of each metabolite. Colored lines indicate the expression dynamics of each metabolite; black lines represent the representative expression of each cluster. PCA, Principal Component Analysis; DAMs, Differentially Accumulated Metabolites.

To investigate the metabolic changes of kiwifruit canes under the infection stress of Psa pathogen, this study selected the phloem of canes from HY and *A. valvata* inoculated with Psa for 5 days as materials, and conducted analysis using UPLC-MS/MS. The metabolic characteristics of each sample were analyzed, and a total of 1229 metabolites were identified (**Supplementary Table S4**). The ring chart of metabolite class composition shows 12 differential metabolite classes, among which flavonoids account for the largest proportion, followed by phenolic acids, and then other classes (**Figure 3A**), including 124 Amino acids and derivatives, 176 phenolic acids, 57 nucleotides and derivatives, 254 flavonoids, 14 quinones, 83 lignans and coumarins, 169 other metabolites, 18 tannins, 89 alkaloids, 80 terpenoids, 66 organic acids, and 99 lipids (**Figure 3A**). Principal component analysis (PCA) of samples under different treatments showed significant metabolic changes in response to Psa infection, with the changes being particularly prominent in *A. valvata*. The first principal component PC1 and the second principal component PC2 explained 49.01% and 8.97% of the total variance (**Figure 3B**). Unsupervised correlation analysis exhibited consistency with the PCA findings, as biological replicates from the same variety subjected to the same treatment showed high similarity (**Figure 3C**). **Figure 3D** presents a heatmap of metabolite contents, with cluster analysis performed on metabolites, which revealed that amino acids and derivatives、alkaloids and lipids accumulated in *A. valvata* under Psa infection. In HY, compared with the ck, 210 differentially DAMs (69 upregulated and 141 downregulated) were identified, in contrast, there were 119 upregulated and 36 downregulated DAMs in *A. valvata*_Psa compared with the ck (**Figure 3E**). Venn diagrams were used to distinguish the common and exclusive metabolites of kiwifruit among different groups after Psa and H_2_O treatment (**Figure 3F**). Our results showed that a relatively large number of DAMs were identified in *A. valvata* and HY under Psa infection. These differences demonstrated that HY and *A. valvata* exhibited distinct metabolite accumulation patterns under psa infection, which may be associated with the differences in their disease resistance capabilities. Of the three groups of differentially expressed metabolites, 31 metabolites were shared between the comparisons of *A. valvata*_Psa_vs_*A. valvata*_ck and *A. valvata*_Psa_vs_HY_Psa (**Figure 3F**). These 31 DAMs mainly included flavonoids, organic acids, terpenoids, and amino acids and their derivatives, among others (**Supplementary Table S5**).

To further understand the metabolic changes during the defense process of different kiwifruit varieties after Psa infection, the k-means clustering algorithm was used to classify 1229 DAMs into 9 distinct clusters (**Supplementary Table S6**). Among these, two clusters of specific metabolites (Cluster 1 and Cluster 6) were significantly induced and accumulated in *A. valvata* upon Psa infection, whereas no significant changes were observed in HY. These metabolites are mainly included flavonoids, terpenoids, lipids, phenolic acids, and alkaloids (**Figure 3G**; **Supplementary Table S6**), which play important roles in plant biotic stress responses, suggesting that these pathways may help *A. valvata* resist pathogen infection to a greater extent.

### 3.4 Combined transcriptome and metabolome analysis

**Figure 4:**
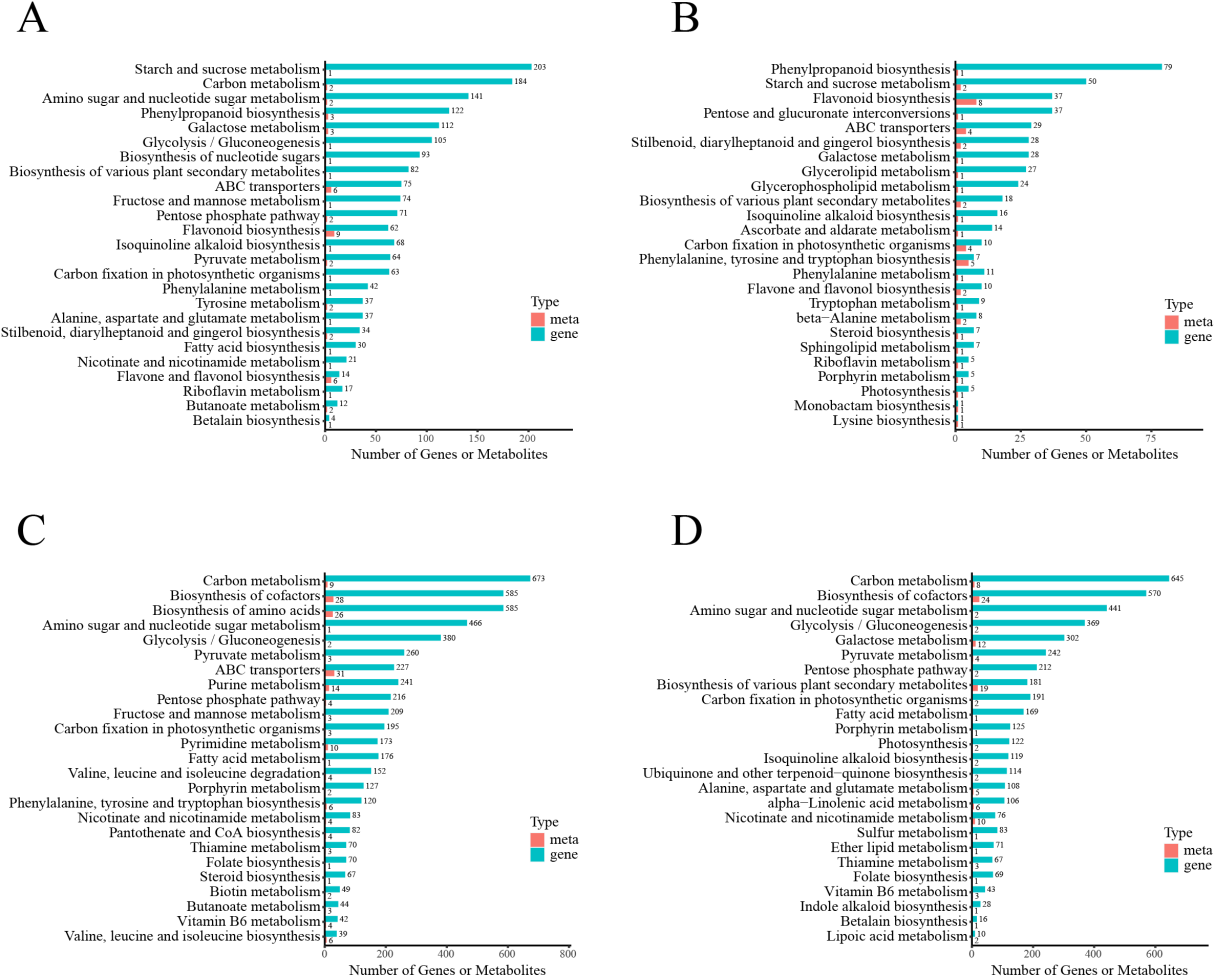
KEGG pathways co-enriched by transcriptome and metabolome. **(A)** Enrichment pathways for the comparison *A. valvata*_Psa_vs_*A. valvata*_ck. **(B)** Enrichment pathways for the comparison HY_Psa_vs_HY_ck. **(C)** Enrichment pathways for the comparison *A. valvata*_ck_vs_HY_ck. **(D)** Enrichment pathways for the comparison *A. valvata*_Psa_vs_HY_Psa. Red columns represent metabolite enrichment, and green columns represent gene enrichment.

To investigate the metabolic and genetic basis underlying *A. valvata* resistance, pathway enrichment analysis was performed across four comparative groups (**Figure 4**). For *A. valvata* under Psa infection versus control, pathways associated with starch and sucrose metabolism (harboring the largest number of annotated genes/metabolites), carbon metabolism, amino sugar and nucleotide sugar metabolism, and phenylpropanoid biosynthesis were prominently enriched (**Figure 4A**). In contrast, HY under Psa infection versus control showed far fewer enriched pathways (phenylpropanoid biosynthesis, starch and sucrose metabolism) with substantially lower counts of annotated molecules (**Figure 4B**). When comparing *A. valvata* and HY under control conditions, pathways including carbon metabolism, biosynthesis of cofactors, and amino sugar and nucleotide sugar metabolism were enriched with abundant genes and metabolites, reflecting inherent metabolic differences between the two varieties in the absence of Psa (**Figure 4C**). Under Psa infection, inter-varietal comparison still enriched pathways like carbon metabolism and amino sugar and nucleotide sugar metabolism (**Figure 4D**), but with a distinct pathway profile from the control condition, indicating Psa infection remodels metabolic divergence between the two varieties. Collectively, *A. valvata* activates more extensive and intensive carbohydrate metabolism and secondary biosynthesis pathways (e.g., phenylpropanoids) upon Psa challenge compared to HY, which may underpin its resistance.

**Figure 5:**
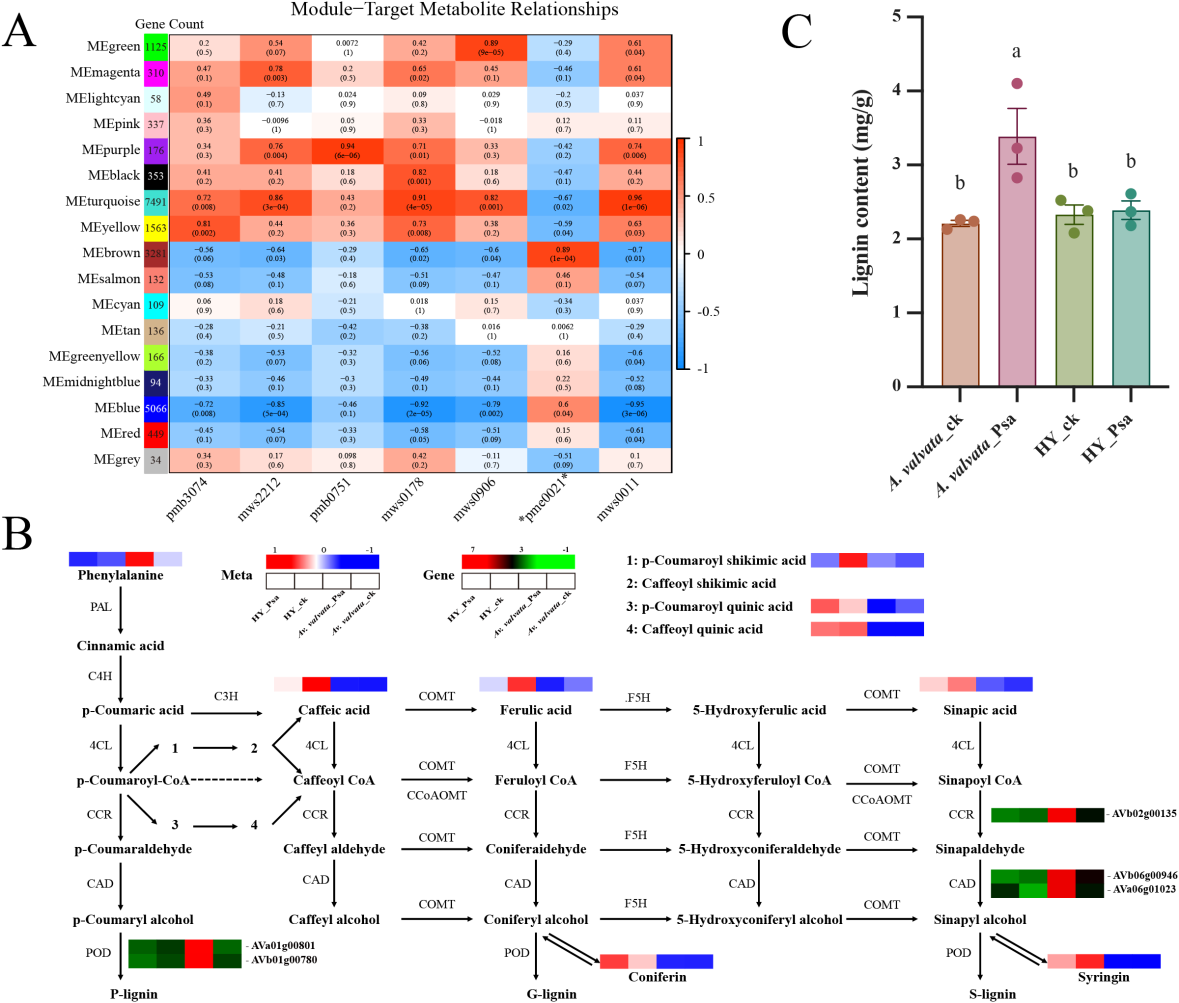
Joint analysis of transcriptome and metabolome under Psa infection. **(A)** WGCNA of correlations between genes and seven metabolites (pmb3074: p-Coumaroylquinic acid; mws2212: Caffeic acid; pmb0751: p-Coumaroyl shikimic acid; mws0178: Caffeoylquinic acid; mws0906: Coniferin; pme0021: L-Phenylalanine; mws0011: Syringin). **(B)** Heatmap of metabolites and genes involved in lignin biosynthesis pathways. **(C)** Quantification of lignin content following Psa inoculation. WGCNA, Weighted Gene Co-Expression Network Analysis, PAL, Phenylalanine ammonia-lyase, C4H, Cinnamate 4-hydroxylase; 4CL, 4-Coumarate-CoA ligase, CCR, Cinnamoyl-CoA reductase; CAD, Cinnamyl alcohol dehydrogenase; POD, Peroxidase; COMT, Caffeic acid O-methyltransferase; CCoAOMT, Caffeoyl-CoA O-methyltransferase; F5H, Ferulate 5-hydroxylase. Data are presented as mean ± SEM (n=3 biological replicates).

### 3.5 WGCNA analysis deciphers variety-specific activation of lignin biosynthesis via key genes in Psa-challenged

In order to further investigate the differential response mechanisms of the two varieties under Psa infection, WGCNA analysis was performed to cluster filtered genes into 17 distinct modules (**Figure 5A**). Based on the KEGG analysis in **Figure 4**, we selected phenylpropanoid biosynthesis metabolites [p-Coumaroylquinic acid (pmb3074), Caffeic acid (mws2212), pmb0751, Caffeoylquinic acid (mws0178), Coniferin (mws0906), L-Phenylalanine (pme0021), Syringin (mws0011)] as phenotypic traits, interestingly, except that the metabolite pme0021 exhibited abundant accumulation in *A. valvata* under Psa treatment, all other metabolites showed higher accumulation in either the HY Psa treatment group or the CK treatment group (**Figure 5B**). And we found that the MEbrown module showed significant correlations with pme0021 metabolites (**Figure 5A**). This module contains 3,281 genes, 2,753 of which were defined as core genes (**Supplementary Table S7**). Further comparison with 1,781 DEGs revealed that 766 genes were common genes (**Supplementary Table S8**). Based on KEGG annotation analysis, one CCR gene (AVb02g00135), two CAD genes (AVa06g01023, AVb06g00946) two POD genes (AVa01g00801, AVb01g00780) were found to be upregulated in *A. valvata*_Psa (**Figure 5B**). Furthermore, AVa01g00801 and AVb01g00780 are classified as peroxidases, which play critical roles in lignin polymerization and the formation of cross-linking structures between lignin, cellulose, and extensins in secondary cell walls. After inoculation, the expression levels of these two lignin-forming genes in *A. valvata* increased significantly. Specifically, compared with HY, the expression levels of AVa01g00801 and AVb01g00780 in *A. valvata* were significantly upregulated by 15-fold and 30-fold, respectively, at 5 dpi. To explore the transcriptional regulation mechanism of these key lignin biosynthetic genes, we further analyzed their promoter regions. Using PlantCARE database, we predicted cis-acting elements in the 2000 bp upstream of TSS of these genes and found that the promoters contained multiple cis-elements specifically bound by WRKY (W-box, TTGACC) and MYB (MYB-binding site) transcription factors (TFs) (**Supplementary Table S10**). For instance, the promoter of AVa01g00801 harbored MYB-binding sites and MBS elements, while AVb02g00135 contained W-boxes, suggesting these genes might be direct targets of WRKY/MYB TFs. These results indicate that the lignin biosynthesis pathway in *A. valvata* was activated, leading to increased lignin synthesis, in contrast, the lignin biosynthesis pathway in HY was predominantly arrested at the stage of producing intermediate products and generating transport/storage forms. This hindered the final formation of mature lignin, leading to a low lignin content and poor disease resistance in HY. Quantification of lignin content assays further confirmed significant lignin accumulation in *A. valvata* canes post-inoculation. As shown in **Figure 5C**, resistant *A. valvata* exhibited a significant increase in lignin content levels after Psa inoculation.

### 3.6 Validation of RNA-seq data by RT-qPCR

**Figure 6:**
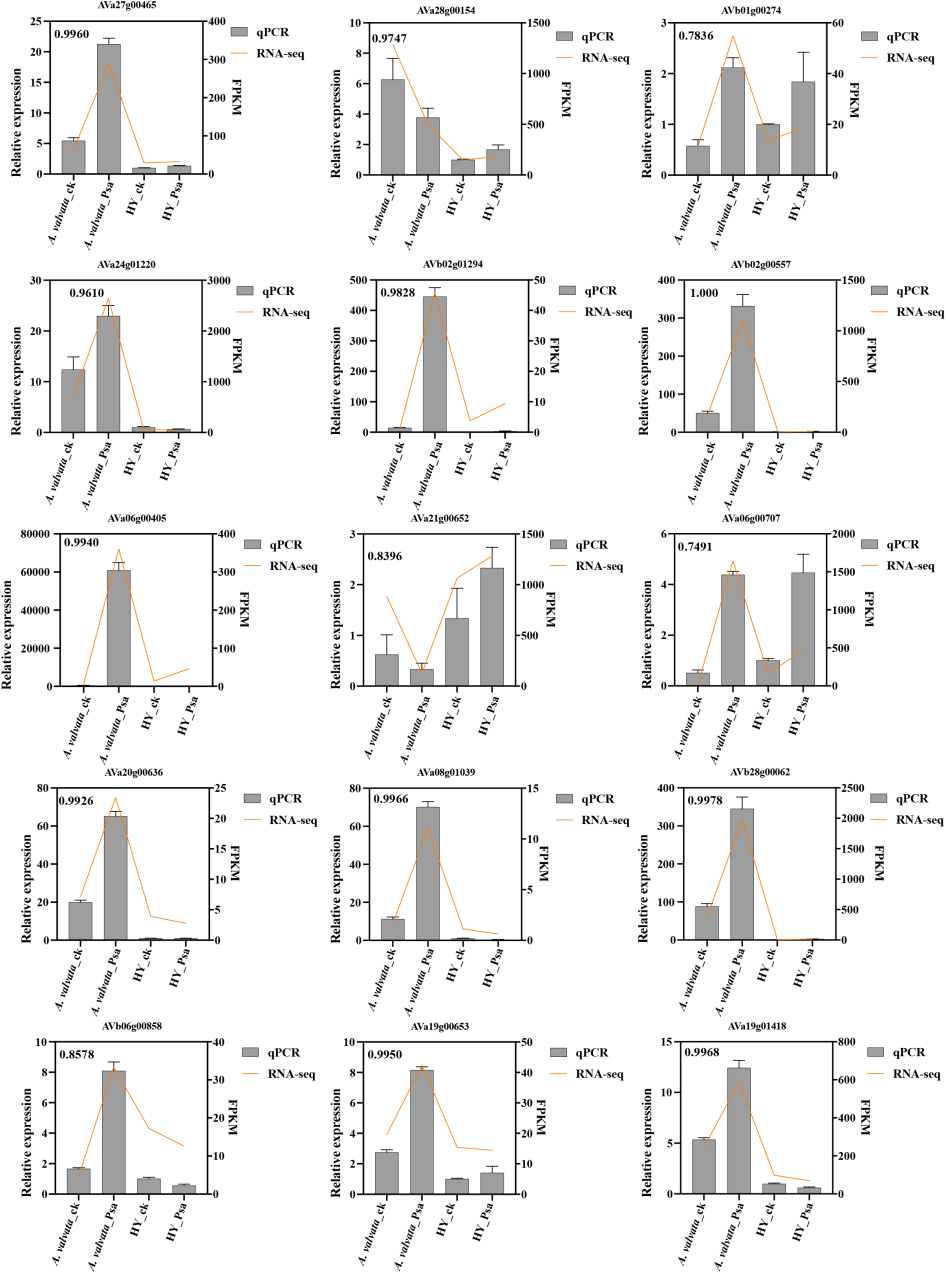
RT-qPCR validation of *A. valvata* and HY samples. The bar chart shows RT-qPCR results, while the line chart presents FPKM values from RNA-seq data. FPKM, Fragments per kilobase of transcript per million fragments mapped. RT-qPCR data are presented as mean ± SEM (n=3 technical replicates).

To validate the accuracy and reliability of the transcriptome data, we randomly selected 15 differentially expressed genes for RT-qPCR analysis (**Figure 6**). We compared the RT-qPCR results with the FPKM values of these genes, calculating the correlation coefficient (defined as r) using a correlation function. The results revealed a strong correlation between the transcriptome data and RT-qPCR outcomes, with an average r of 0.94, confirming the reliability of the transcriptome dataset.

## 4 Discussion

### 4.1 Transcriptomic differences reveal the molecular divergence in variety-specific resistance

To elucidate the genetic basis of Psa resistance in kiwifruit, this study compared the phenotypes of the resistant variety *A. valvata* and the susceptible variety HY after Psa inoculation (**Figure 1**), revealing a striking divergence in their resistance phenotypes. Through transcriptomic analysis of two kiwifruit varieties, this study revealed distinct gene expression regulatory networks. Upon Psa infection, *A. valvata* prominently activated defense-responsive pathways, including Plant-pathogen interaction, Plant hormone signal transduction, and MAPK signaling pathway (**Figure 2**), which are well-documented to mediate rapid pathogen recognition and immune activation in diverse plant species (Zhang et al., 2024a). A total of 765 variety-specific DEGs were identified in *A. valvata* (**Figure 2C**), and these DEGs were significantly enriched in disease resistance-related biological processes. More importantly, the finding that the 1,781 shared DEGs were enriched in the same core defense pathways as the *A. valvata*-specific DEGs, suggestting that resistance is mediated by a pre-configured transcriptional network that integrates both common and variety-specific immune components. PR proteins and RKs key gene families are known to mediate plant immunity (van Loon et al., 2006; Zhang et al., 2024b; Zipfel et al., 2006), notably, the upregulated DEGs in *A. valvata* included those important genes (**Supplementary Table S3**). PR proteins inhibit pathogen colonization by disrupting microbial cell walls or suppressing toxin activity (Dong, 2004; van Loon et al., 2006), while RKs function as crucial sensors for pathogen-associated molecular patterns (PAMPs) or effector molecules, triggering early immune responses (Zipfel et al., 2006). In contrast, only 106 species-specific DEGs were detected in the susceptible HY variety (**Figure 2C**), and these genes were highlighted two major pathways: starch and sucrose metabolism and phenylpropanoid biosynthesis, with no significant association with defense signaling (**Figure 2D**). Starch and sucrose metabolism regulates the conversion between sucrose and starch. Sucrose, the main form of photosynthetic product transport, and starch is the main storage carbohydrate. This pathway supplies basic energy and carbon skeletons for plant physiological processes but reflects a general metabolic adjustment rather than a specialized defense response (Lastdrager et al., 2014). Phenylpropanoid biosynthesis is essential for producing secondary metabolites that underpin plant physical and chemical defenses (Cui et al., 2024; Lü et al., 2025; Singh, 2025). Despite this, the limited number of DEGs and low enrichment level in these basal metabolic pathways in HY indicate that during Psa challenge, this genotype relies more on preliminary resource allocation. This deficiency not only lies in inefficient resource utilization but also in the lack of a robust transcriptional defense response, ultimately undermining HY’s ability to counteract Psa invasion and leading to its high susceptibility.

The substantial difference in DEG number and functional enrichment between the two varieties further supports the notion that *A. valvata* possesses a preconfigured or rapidly inducible immune regulatory network, whereas HY lacks the genetic machinery to mount an effective defense against Psa. This observation aligns with previous transcriptomic studies on Psa-kiwifruit interactions, which highlighted that resistant genotypes typically exhibit broader and more intense transcriptional reprogramming in defense pathways compared to susceptible counterparts (Qin et al., 2022; Wang et al., 2021). Transcription factors are central regulators of defensive responses in plants (Fan et al., 2024; Zhao et al., 2025; Zhou et al., 2024). Our integrated analysis identified members of the WRKY, MYB, and NAC families among the core DEGs overlapping with the phenylpropanoid-related co-expression module (**Supplementary Table S8**). Most of these TFs were significantly up-regulated upon Psa infection in *A. valvata*; these TFs are strong candidates for orchestrating the observed defense pathway activation, given their well-established roles in regulating secondary metabolism and immune responses in other species. Their specific regulatory roles in mediating Psa resistance remain to be elucidated, representing a key direction for future research.

### 4.2 Metabolomic features and differential metabolite accumulation underpin Psa resistance divergence between *A. valvata* and HY

Metabolomic analysis using UPLC-MS/MS further uncovered the metabolic basis of Psa resistance in *A. valvata*, with distinct metabolite accumulation patterns observed between the resistant and susceptible varieties (**Figure 3**). The identified metabolites were predominantly from classes with known defensive functions, such as flavonoids and phenolic acids (**Figure 3A**), indicating a metabolic landscape primed for defense responses. Notably, the specifically induced Clusters 1 and 6 in *A. valvata* post-infection are enriched (**Figure 3G**). Cluster 1 is dominated by flavonoids, terpenoids, alkaloids, and phenolic acids, along with organic acids and nucleotides/derivatives; while terpenoids and alkaloids directly inhibit microbial growth by disrupting membrane integrity or enzyme activity (Dixon and Paiva, 1995; Mierziak et al., 2014), these compounds collectively contribute to cell wall reinforcement, reactive oxygen species (ROS) scavenging, and direct antimicrobial activity (Babenko et al., 2019; Gu et al., 2021). Cluster 6 is marked by an overwhelming abundance of flavonoids, accompanied by high representation of nucleotides/derivatives, amino acids/derivatives, and lipids. While flavonoids in this cluster likely exert conserved defensive functions, the enrichment of nucleotides and amino acids may support rapid biosynthesis of defense-related proteins and nucleic acids, sustaining the prolonged immune response against Psa colonization (Aliferis et al., 2014). The co-enrichment of these functional metabolite classes in both clusters underscores a coordinated metabolic reprogramming strategy deployed by the resistant genotype to counteract pathogen attack. PCA showed that Psa infection induced significant metabolic shifts, especially in *A. valvata* (**Figure 3B**). A heatmap of metabolite abundances further specified that *A. valvata* uniquely accumulated amino acids/derivatives, alkaloids, and lipids upon Psa infection, which changes that were not observed in HY (**Figure 3D**), reflecting a defense-oriented metabolic reprogramming exclusive to the resistant variety.

Striking differences in DAMs between the two species further supported their divergent resistance phenotypes. The metabolic response in HY was characterized by a net downregulation upon Psa challenge (**Figure 3E**), suggesting a disrupted or suppressed metabolic state associated with susceptibility. In contrast, *A. valvata* exhibited a net upregulation of metabolites, indicating a proactive defense mobilization. Venn diagram analysis further pinpointed 31 DAMs shared between key comparisons (**Figure 3F**); these core metabolites, including flavonoids and amino acid derivatives, likely function as key mediators of resistance.

### 4.3 Integrated multi-omics analysis uncovers the systematic regulatory network of resistance

Integrating transcriptomic and metabolomic data enabled a systematic dissection of the regulatory networks driving *A. valvata*’s resistance to Psa, revealing a coordinated interplay between carbohydrate metabolism, secondary biosynthesis, and cell wall reinforcement.

Pathway enrichment analysis across four comparative groups first highlighted species-specific metabolic reprogramming (**Figure 4**). In *A. valvata* (infected vs. control), pathways critical for defense were prominently enriched, including starch and sucrose metabolism, which annotated with the largest number of genes and metabolites, followed by carbon metabolism, amino sugar and nucleotide sugar metabolism, and phenylpropanoid biosynthesis (**Figure 4A**). While these pathway enrichments were identified, a deeper mechanistic analysis of starch and sucrose metabolism, as well as phenylpropanoid biosynthesis, remains to be fully explored. For starch and sucrose metabolism, *A. valvata*’s active redirection of carbohydrate flux likely involves key enzymes such as sucrose synthases (SUS) and invertases (INV), which hydrolyze sucrose into glucose and fructose to fuel both energy production and precursor supply for secondary metabolism. This metabolic rewiring may be coordinated by transcription factors that simultaneously upregulate SUS/INV genes and downstream phenylpropanoid enzymes, the regulatory pattern that facilitates the efficient supply of precursors for defense metabolite production (Ferri et al., 2011; Li et al., 2021). Regarding phenylpropanoid biosynthesis, the accumulation of L-phenylalanine in *A. valvata* and the upregulation of CCR, CAD, and POD genes suggest a tightly regulated pathway where the rate-limiting step (PAL) and downstream lignin polymerization enzymes are synergistically activated. Further, the differential accumulation of intermediate metabolites between *A. valvata* and HY implies species-specific metabolic checkpoints, *A. valvata* may prioritize lignin monomer synthesis, while HY accumulates intermediates due to impaired downstream polymerization, a hypothesis that could be tested via targeted metabolite profiling and enzyme activity assays (Gao et al., 2025). In tomato, overexpression of *SlβCA3* enhances the resistance of plants to Pst DC3000, which may be related to the role of sugar metabolism and signaling in plant immunity (Fang et al., 2022). In this study, *A. valvata* actively redirects carbohydrate metabolism, starch hydrolysis provides glucose as both energy source and carbon skeleton for secondary metabolite synthesis, while carbon metabolism sustains the high metabolic demand of defense responses. In contrast, HY (infected vs. control) showed only two weakly enriched pathways (phenylpropanoid biosynthesis and starch/sucrose metabolism) with far fewer annotated molecules (**Figure 4B**), suggesting that it lacks the breadth of pathway activation required for effective metabolic reprogramming. Under control conditions, inter-varietal comparisons enriched pathways like carbon metabolism and amino sugar metabolism (**Figure 4C**), reflecting inherent genetic differences that prime *A. valvata* for rapid defense activation. Under Psa infection, these inter-varietal pathway differences were further remodeled (**Figure 4D**), confirming Psa specifically amplifies metabolic divergence between resistant and susceptible genotypes.

WGCNA further refined this regulatory network by clustering filtered genes into 17 modules, with the MEbrown module showing a significant correlation with phenylpropanoid metabolites (L-Phenylalanine, pme0021) (**Figure 5A**). Notably, L-Phenylalanine a rate-limiting precursor of phenylpropanoid biosynthesis, accumulated abundantly in infected *A. valvata*, while other phenylpropanoid metabolites were more abundant in HY (infected or control) (**Figure 5B**). The MEbrown module contained a set of core genes, a significant portion (766) of which overlapped with the previously identified defense-associated DEGs (**Supplementary Table S8**). This overlap defines a high-confidence, resistance-related co-expression gene set. KEGG annotation of this set revealed key lignin biosynthetic genes, one CCR (AVb02g00135), two CAD (AVa06g01023, AVb06g00946), and two POD (AVa01g00801, AVb01g00780) (**Figure 5B**). Among them, the POD genes were upregulated by 15-fold and 30-fold in infected *A. valvata* compared to HY, respectively; they play pivotal roles in lignin polymerization and cross-linking lignin with cellulose/extensins, directly strengthening the cell wall (Cosgrove, 2005).

To further decipher the regulatory logic of the MEbrown module in linking transcriptional control to phenylpropanoid metabolism and resistance, we focused on key TFs enriched in this module, specifically WRKY and MYB family members, which are well-documented regulators of plant secondary metabolism and defense responses (Wang et al., 2024; Yang et al., 2024). The 766 core DEGs in the MEbrown module identified 17 WRKY and 12 MYB genes, whose expression patterns were significantly correlated with the upregulation of lignin biosynthetic genes (**Supplementary Table S8**). Existing literature provides strong support for the regulatory role of these TFs in lignin biosynthesis. For instance, Wang et al. (2024) demonstrated that PbWRKY24 in pear directly binds to the W-box cis-element (TTGACC/T) in the promoter of PbPRX4 (a POD homolog), activating its transcription to enhance lignin accumulation contributes to russet skin formation in pear fruits. Notably, StMYB168 and StWRKY20 have been shown to form a synergistic complex to activate CAD and PAL genes, further amplifying lignin monomer synthesis during potato wound healing (Yang et al., 2024). Based on these precedents, we hypothesize that WRKY and MYB TFs in the MEbrown module of *A. valvata* may specifically bind to the W-box motifs in the promoters of CCR gene and CAD genes, as predicted by cis-element analysis (**Supplementary Table S10**), to amplify their transcriptional output. This TF-mediated activation is inferred to accelerate the conversion of L-phenylalanine into mature lignin, rather than intermediate phenylpropanoid metabolites, thereby strengthening the cell wall barrier against Psa invasion (Gao et al., 2025; Li et al., 2025). This transcriptional regulatory cascade explains the distinct metabolic phenotypes between *A. valvata* and HY, while HY accumulates non-functional phenylpropanoid intermediates due to insufficient TF-mediated activation of downstream lignin biosynthetic genes, *A. valvata* efficiently channels metabolites into lignin synthesis via coordinated WRKY/MYB regulation. This link between TF activity, targeted gene expression, and metabolic flux rerouting directly underpins the resistance phenotype of *A. valvata*.

It is important to acknowledge that while our integrated multi-omics analysis establishes strong correlations between the upregulation of CCR, CAD, and POD genes, the accumulation of lignin-related metabolites, and *A. valvata*’s Psa resistance, this study lacks direct functional validation (e.g., gene knockout or overexpression experiments) to definitively confirm the causal roles of these genes and pathways. To address this, future work should employ genetic approaches such as CRISPR/Cas9-mediated gene knockout or overexpression systems to functionally validate the roles of core lignin biosynthetic genes and key transcription factors in Psa resistance. Additionally, complementation assays and enzyme activity analyses could further elucidate the biochemical mechanisms underlying lignin polymerization and cell wall strengthening during *A. valvata*’s defense response.

This transcriptional regulation of lignin biosynthesis was corroborated by metabolomic and biochemical data. *A. valvata* showed significantly higher lignin accumulation post-inoculation (**Figure 5C**), while HY’s lignin pathway was arrested at intermediate products, limiting mature lignin formation and resulting in fragile cell walls that allowed Psa colonization of phloem (**Figure 5B**). Additionally, k-means clustering of 1229 DAMs identified Clusters 1 and 6, specifically induced in infected *A. valvata*, which included lignin precursors and flavonoid derivatives (**Figure 3G**). This further confirms that *A. valvata*’s resistance arises from coordinated metabolite accumulation and cell wall strengthening. Beyond the mechanistic insights into Psa resistance, our study underscores the enormous potential of wild *Actinidia* germplasm represented by *A. valvata* as a reservoir of elite resistance traits for kiwifruit breeding programs. Unlike commercial cultivars such as *A. chinensis* ‘Hongyang’ that have undergone long-term artificial selection and thus possess a narrow genetic base, wild kiwifruit species have evolved diverse and robust defense strategies against biotic stresses during natural adaptation. The key lignin biosynthesis-related genes and their specific regulatory networks identified in *A. valvata* not only explain its superior Psa resistance, but also provide a set of reliable molecular markers for marker-assisted selection (MAS). Incorporating these markers into breeding pipelines can significantly shorten the breeding cycle, enabling rapid screening of resistant progenies from hybrid populations and accelerating the development of Psa-resistant commercial cultivars. However, the use of a single time point also has limitations: it cannot capture the dynamic changes in the Psa-kiwifruit interaction before or after 5 dpi, such as the initial recognition phase (1–3 dpi) or the late necrosis phase (7–10 dpi), which warrants further time-course experiments in future studies.

## 5 Conclusion

This study delineated the core resistance mechanisms of *A. valvata* against Psa via integrated phenotypic, transcriptomic, and metabolomic analyses, providing a clear framework for understanding kiwifruit’s Psa defense. *A. valvata* triggers a strong transcriptional defense upon Psa infection, with DEGs enriched in core immune pathways (plant-pathogen interaction, hormone signaling, MAPK), while susceptible HY only shows minimal basal metabolic changes. *A. valvata* accumulates defense secondary metabolites (flavonoids, terpenoids, lignin precursors) and strengthens cell walls by upregulating lignin biosynthetic genes, including POD, CCR and CAD. In contrast, HY’s lignin synthesis is impaired, leading to fragile cell walls. Integrated multi-omics provides insights into *A. valvata*’s coordinated reprogramming of carbohydrate and secondary metabolism, which may form a hierarchical network: carbohydrate metabolism provides energy/precursors, phenylpropanoid biosynthesis generates defense compounds, and WGCNA’s MEbrown module are suggested to drive lignin polymerization. HY’s susceptibility is potentially associated with deficiencies in all three tiers.

These results identify key candidate genes and pathways for kiwifruit disease-resistant breeding. Future work should focus on validating core genes via CRISPR/Cas9 or overexpression, conducting field trials to verify resistance stability, and identifying regulatory transcription factors, laying a foundation for developing Psa-resistant commercial varieties.

## Data Availability

The data presented in the study are deposited in the NCBI repository, accession number PRJNA1420270.
